# Supplementary figures and images for: Developing a 670k genotyping array to tag ~2M SNPs across 24 horse breeds
Source: BMC Genomics. 2017 Jul 27;18:565. doi: 10.1186/s12864-017-3943-8 (PMC5530493; doi:10.1186/s12864-017-3943-8)

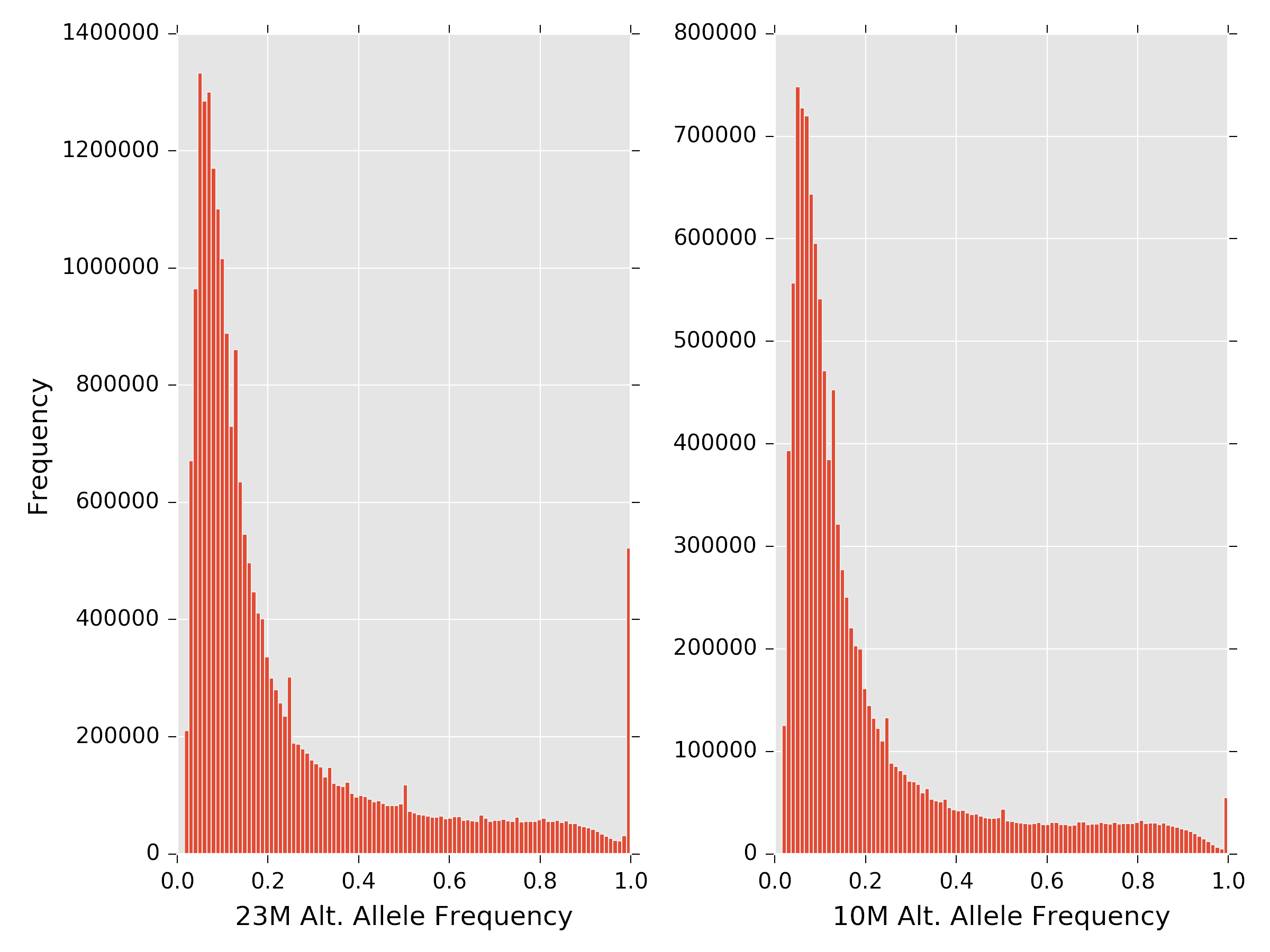

Supplement: Supplementary file 9 — Figure S1. Alternate allele frequency for 23 M and 10 M SNP sets. Histograms show the alternative allele frequency for the ~23 million SNPs discovered by WGS and the ~10 million SNPs that were compatible with array design (see Table 1). A high number of SNPs were observed at 100% alternate allele frequency in a sample cohort that included deep sequencing (see Additional file 1: Table S1) of Twilight (the horse used for the reference genome) indicating likely Sanger sequence errors. (PNG 87 kb) [file 12864_2017_3943_MOESM9_ESM.png]

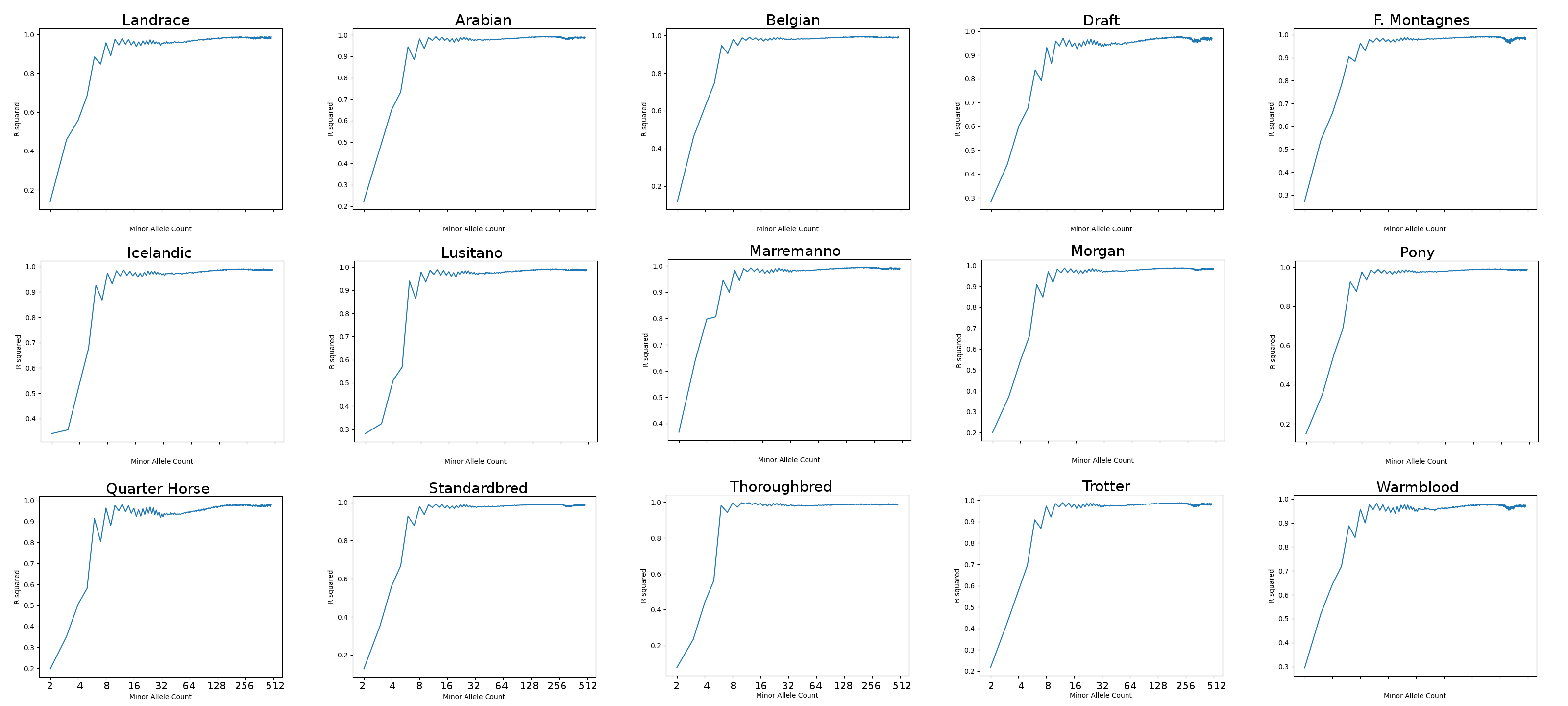

Supplement: Supplementary file 10 — Figure S2. The effects of minor allele on imputation accuracy. SNPs were binned by minor allele count of the marker in the reference panel for each tagging breed group. SNPs were masked down to the 670k set then imputed up to ~2M. For SNPs in each minor allele count bin, a Pearson correlation was calculated between the imputed minor allele dosage and the true minor allele dosage. The x-axis in each panel are on a log scale to show the relationship for low minor allele count SNPs. (PNG 264 kb) [file 12864_2017_3943_MOESM10_ESM.png]

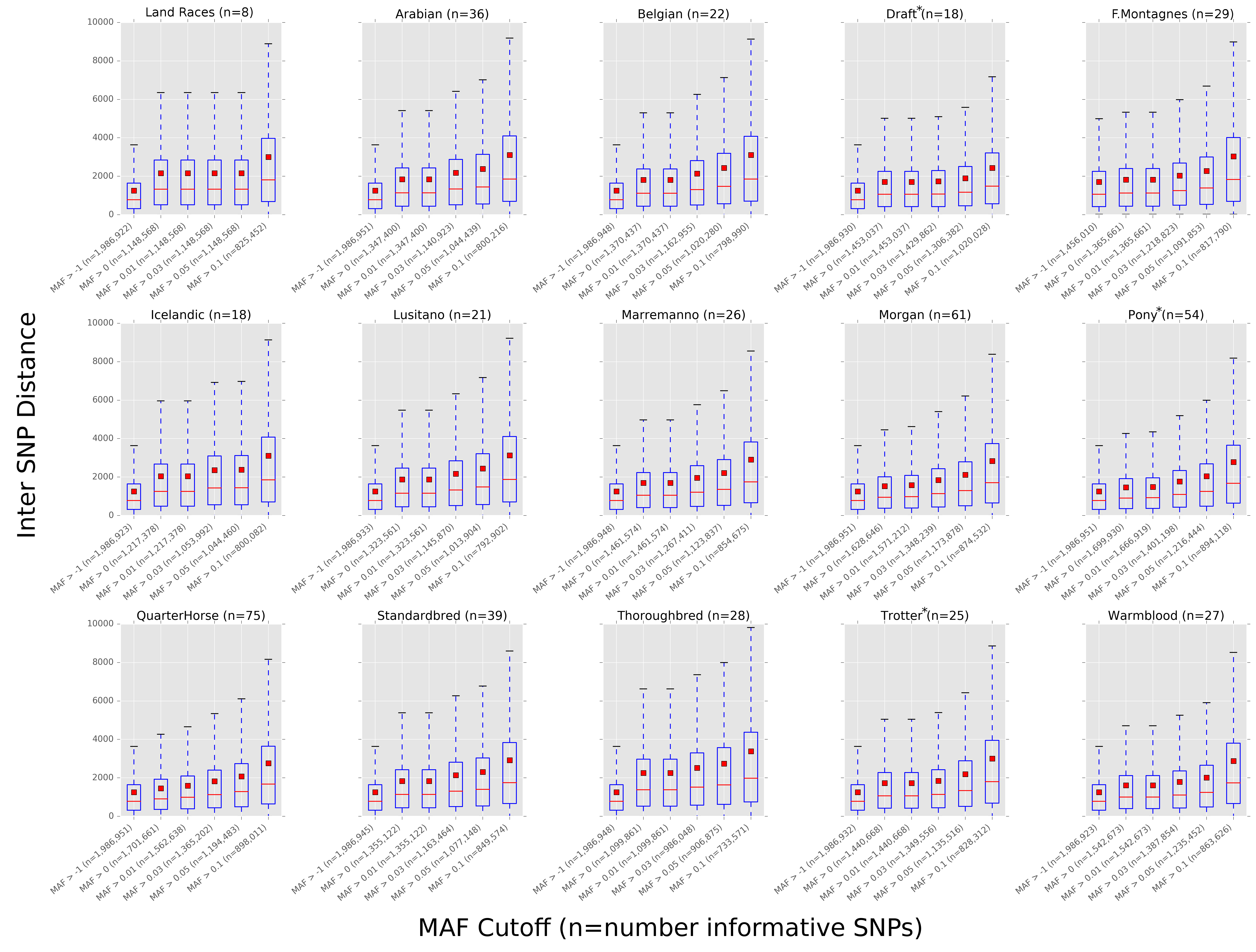

Supplement: Supplementary file 11 — MNEc2M Inter-SNP distance and informativeness by breed. Distance between SNPs on the MNEc2M array were calculated by breed group (See Additional file 5: Table S5) at various minor allele frequency cutoffs (0, 0.01, 0.03, 0.05, 0.10). Breed groups with asterisk (*) indicate a combination of studbook breeds. (PNG 1152 kb) [file 12864_2017_3943_MOESM11_ESM.png]

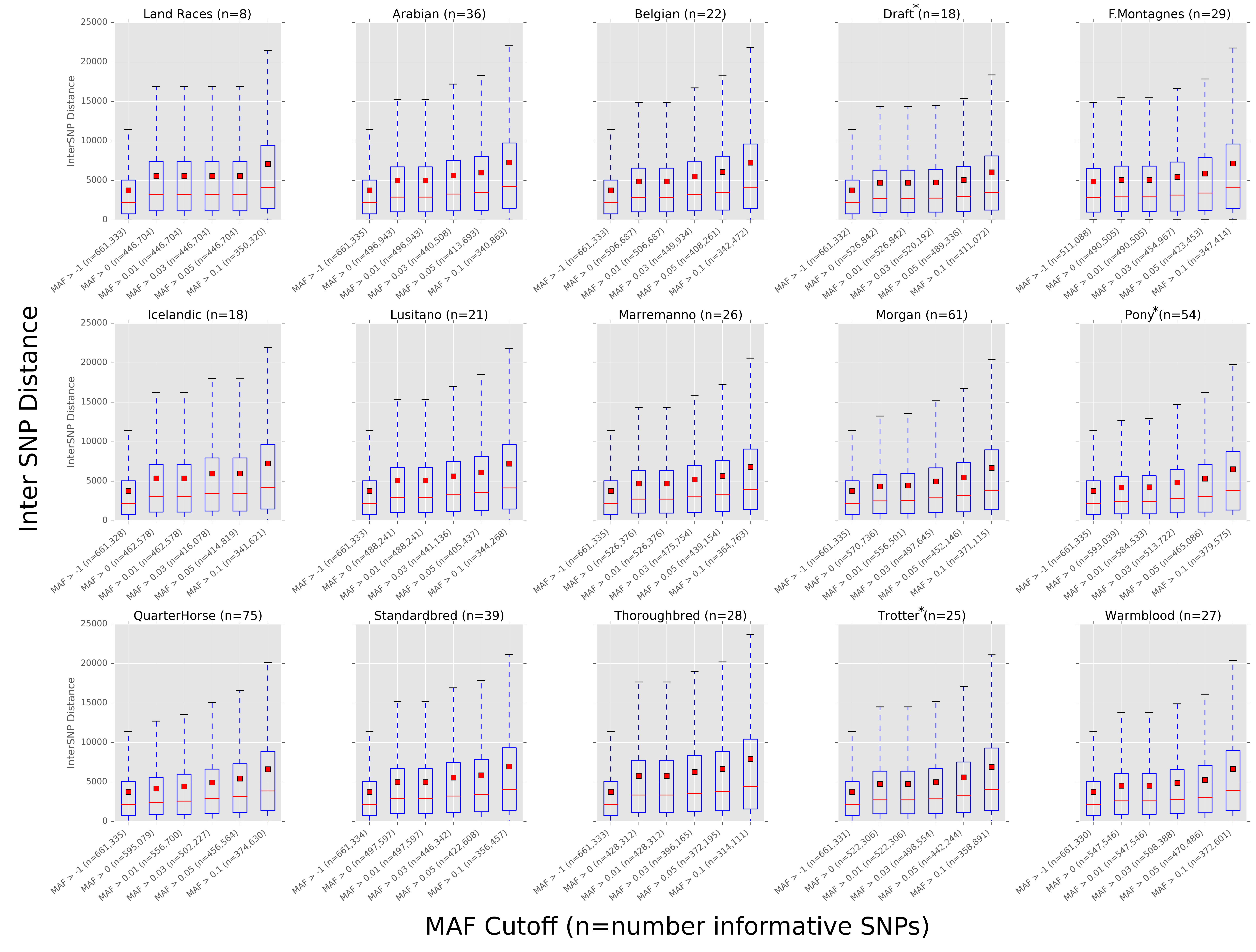

Supplement: Supplementary file 12 — Figure S4. MNEc670k Inter-SNP distance and informativeness by breed. Distance between SNPs on the MNEc670k array were calculated by breed group (Additional file 5: Table S5) at various minor allele frequency cutoffs (0, 0.01, 0.03, 0.05, and 0.10). Breed groups with asterisk (*) indicate a combination of studbook breeds. (PNG 1117 kb) [file 12864_2017_3943_MOESM12_ESM.png]

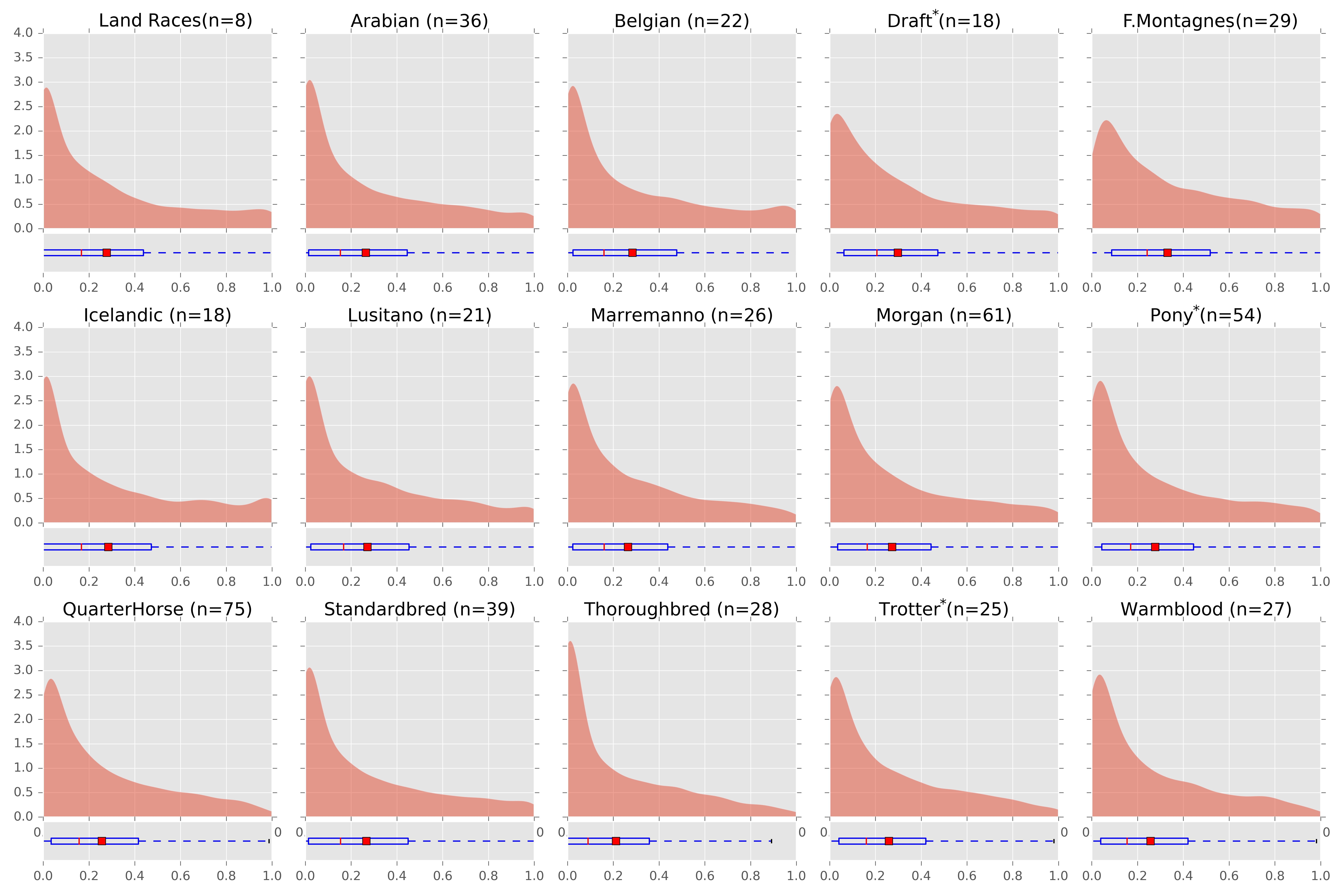

Supplement: Supplementary file 13 — MNEc670k Breed Specific Alternate Allele Frequency. Alternate allele frequencies from variants present on the MNEc670k chip were split by breed group. Samples (WGS + SNP) were split into 15 tagging breed groups (See Additional file 5: Table S5). Breed groups with asterisk (*) indicate a combination of studbook breeds. (PNG 814 kb) [file 12864_2017_3943_MOESM13_ESM.png]

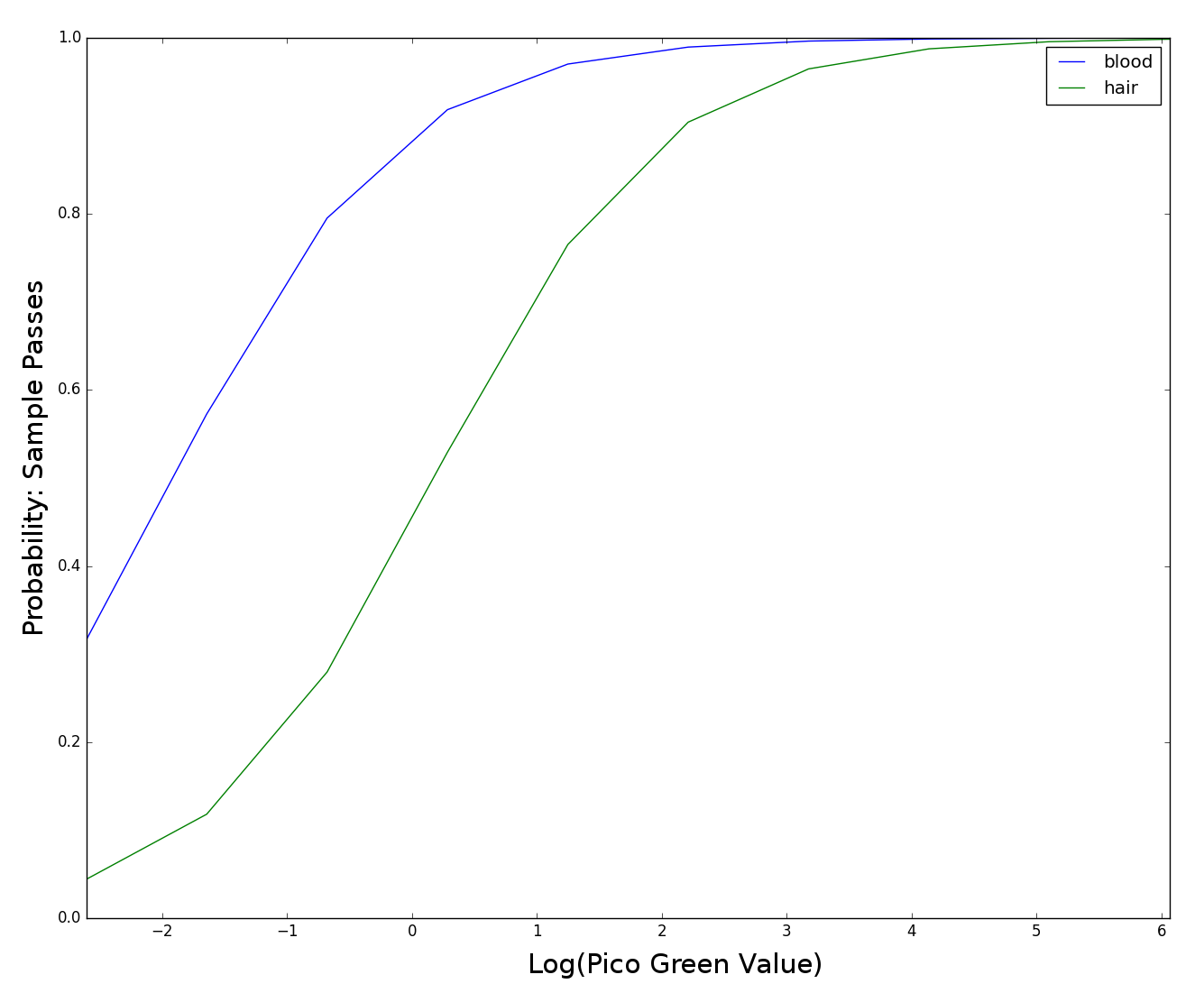

Supplement: Supplementary file 14 — Logistic regression of DNA Sample success. A logistic regression was fit, predicting the probability of a sample passing quality control using DNA concentration (Pico Green) and sample source as independent variables. (PNG 60 kb) [file 12864_2017_3943_MOESM14_ESM.png]
